# RDD2020: An annotated image dataset for automatic road damage detection using deep learning

**DOI:** 10.1016/j.dib.2021.107133

**Published:** 2021-05-12

**Authors:** Deeksha Arya, Hiroya Maeda, Sanjay Kumar Ghosh, Durga Toshniwal, Yoshihide Sekimoto

**Affiliations:** a Center for Transportation Systems, Indian Institute of Technology Roorkee, 247667, India; b Institute of Industrial Science, The University of Tokyo, 4-6-1 Komaba, Tokyo, Japan; c Department of Civil Engineering, Indian Institute of Technology Roorkee, 247667, India; d Department of Computer Science & Engineering, Indian Institute of Technology Roorkee, 247667, India

**Keywords:** Road damage dataset, Deep learning, Structural health monitoring, Road infrastructure, Automatic road condition monitoring, Smartphone-based road damage detection and classification, Pavement surface condition assessment, Crack recognition, Image, Quantification, Data qualification

## Abstract

This data article provides details for the RDD2020 dataset comprising 26,336 road images from India, Japan, and the Czech Republic with more than 31,000 instances of road damage. The dataset captures four types of road damage: longitudinal cracks, transverse cracks, alligator cracks, and potholes; and is intended for developing deep learning-based methods to detect and classify road damage automatically. The images in RDD2020 were captured using vehicle-mounted smartphones, making it useful for municipalities and road agencies to develop methods for low-cost monitoring of road pavement surface conditions. Further, the machine learning researchers can use the datasets for benchmarking the performance of different algorithms for solving other problems of the same type (image classification, object detection, etc.). RDD2020 is freely available at [1]. The latest updates and the corresponding articles related to the dataset can be accessed at [2].

**Specifications Table**

**Table:** 

Subject	Computer Vision and Pattern Recognition,
	Computer Science Applications,
	Artificial Intelligence
Specific subject area	Smartphone-based Road Damage Detection and Classification using Image Processing and Deep Learning
Type of data	2D-RGB Images (.jpg), Annotation Files (.xml), Label Map(.pbtxt)
How data were acquired	Road images (.jpg) were collected using a vehicle-mounted smartphone, moving at an average speed of about 40Km/h. XML files were created using the LabelImg tool to annotate the road damages present in the images.
Data format	Raw images– (.jpg)
	Annotation Files – (.xml) in Pascal VOC Format [3]
	Label Map(.pbtxt)
Parameters for data collection	The road images were collected in daylight, considering a wide variety of weather and illuminance conditions while capturing the images.
Description of data collection	A smartphone application was created to collect the road images once per second from a moving vehicle. For Japan and Czech Republic, the smartphone LG Nexus 5X was used. For India, Samsung Galaxy J6 was used to host the application.
Data source location	Country: India, Japan, Czech Republic
Data accessibility	Repository name: Mendeley
	Data identification number: 10.17632/5ty2wb6gvg.1
	Direct URL to data: http://dx.doi.org/10.17632/5ty2wb6gvg.1

**Value of the Data**

• The RDD2020 data provides the basis for smartphone-based automatic road damage detection and is useful for municipalities and road agencies for low-cost monitoring of road conditions.
• RDD2020 data is valuable for developing new deep convolutional neural network architectures or modifying the existing architectures to improve the performance of the network. Researchers can use the data to train, validate, and test the algorithms for detecting road damages in multiple countries.
• Currently, the data contains the road images from three countries (India, Japan, and the Czech Republic). Researchers or pavement engineers may utilize the data for other countries by following the procedure given in the research article [4].
• At present, the data supports the detection and classification of road cracks (longitudinal, transverse, and alligator) and potholes. It can be further extended to cover other damage categories.
• Machine learning researchers can use the datasets for benchmarking the performance of different algorithms for solving other problems of the same type (image classification, object detection, etc.).
• RDD2020 data can be used to organize data challenges. For instance, the Global Road Damage Detection Challenge (GRDDC'2020), organized as an IEEE Big Data Cup in 2020, utilized the dataset RDD2020 to evaluate the road damage detection models proposed by participants [5,6].

## 1 Data Description

**Fig. 1:**
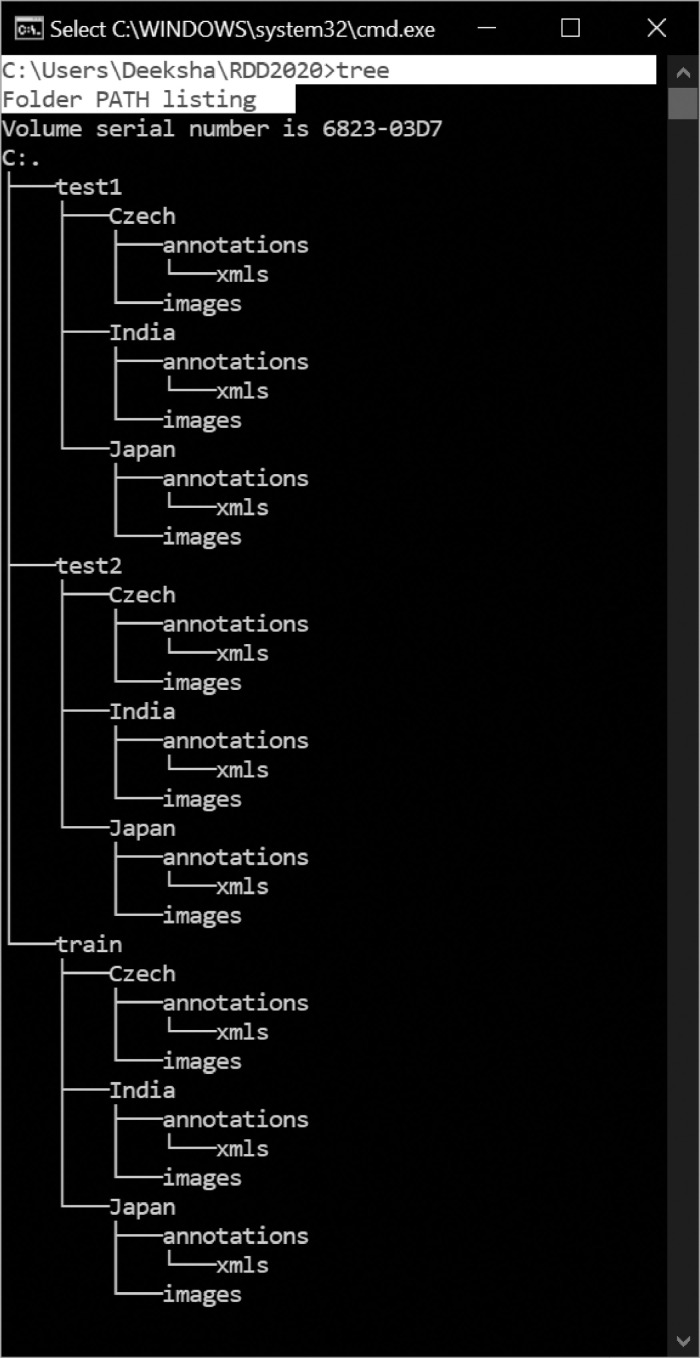
The directory structure.

The RDD2020 image dataset contains 26,336 road images collected from India, Japan, and the Czech Republic, with more than 31,000 instances of road damages. The dataset contains annotation for four damage categories: Longitudinal Cracks(D00), Transverse Cracks(D10), Alligator Cracks(D20) and Potholes(D40). The directory structure for the data is shown in Fig. 1.

**Fig. 2:**
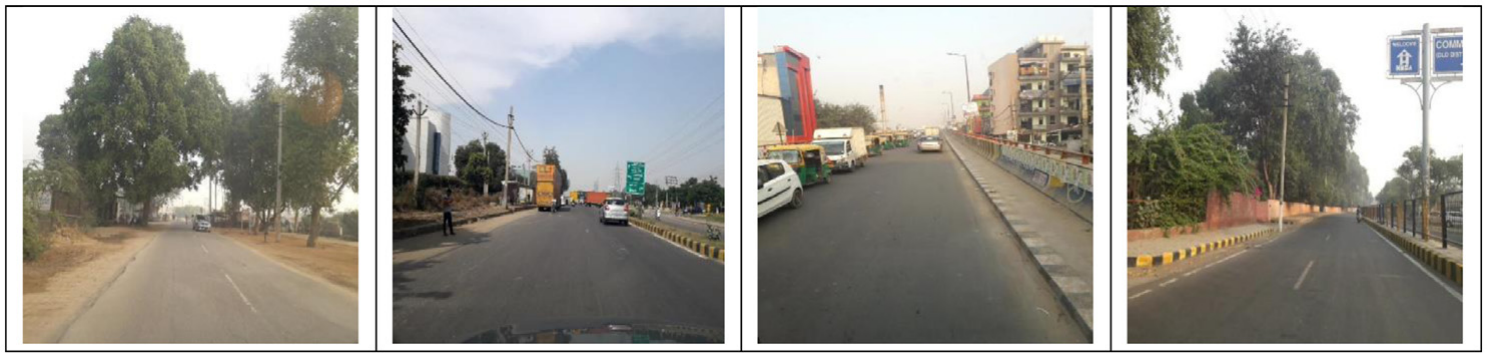
Sample Images from India.

**Fig. 3:**
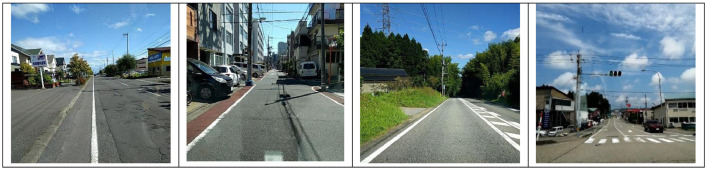
Sample Images from Japan.

**Fig. 4:**
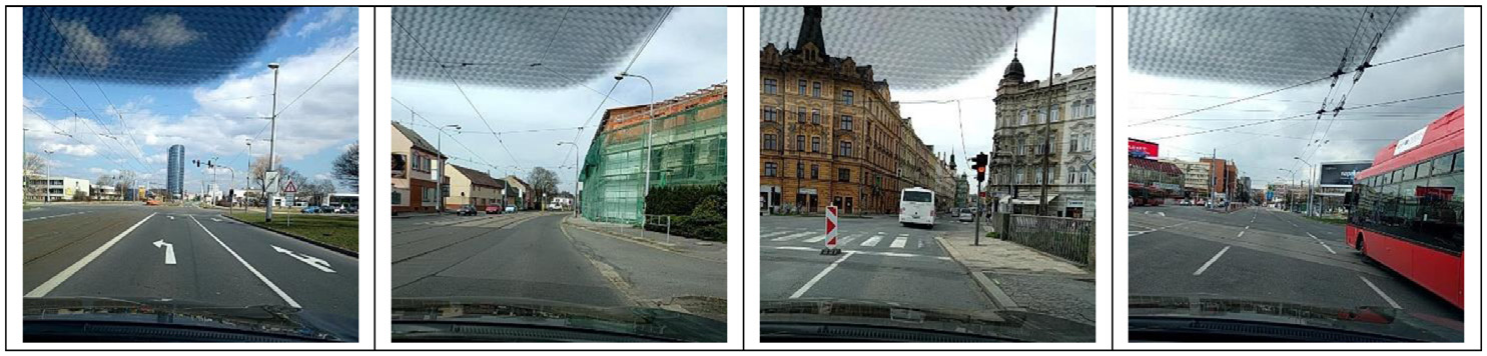
Sample Images from the Czech Republic.

The data has been divided into three subsets: train, test1, and test2. The train set further contains three subdirectories: India, Japan, and Czech. Each sub-directory consists of images and annotations collected from the respective country. The directory images for Japan and Czech, respectively, contain 10,506 and 2829 image files(.jpg) of the resolution 600 × 600 pixels. For India, the directory 'images' includes 7706 images of resolution 720 × 720. The sample images from India, Japan, and Czech are shown in Figs. 2, 3, and 4, respectively.

**Fig. 5:**
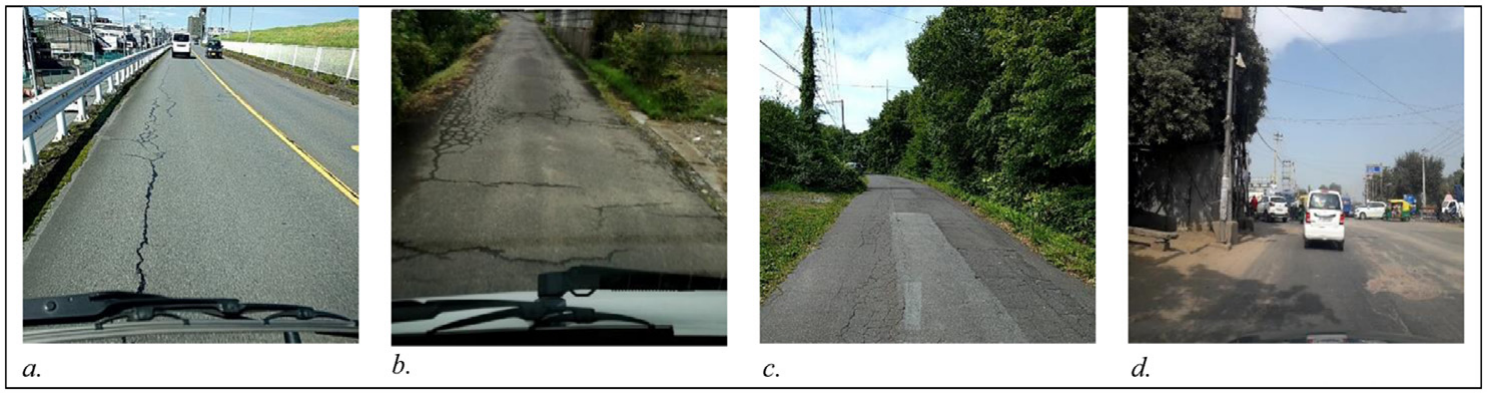
Sample images for road damage categories considered in the data. a. Longitudinal Crack (D00) b. Transverse Crack (D10) c. Alligator Crack(D20) d. Pothole(D40).

The annotation directory contains the corresponding XML files in PASCAL VOC format [3]. It includes the information of the road damage label and its location coordinates in the image. The damage labels mainly cover the four damage categories as mentioned earlier. The corresponding sample images are shown in Fig. 5.

Additionally, some extra damage categories are included for images from Japan to maintain consistency with the previous versions of the dataset, details of which are provided in the research article [4]. Further, it is worth noting that the dataset also contains road images with no aforementioned damage instances present on the pavement surface. These images of un-cracked roads have been included to facilitate false positive detections by models developed for detecting road damages.

**Fig. 6:**
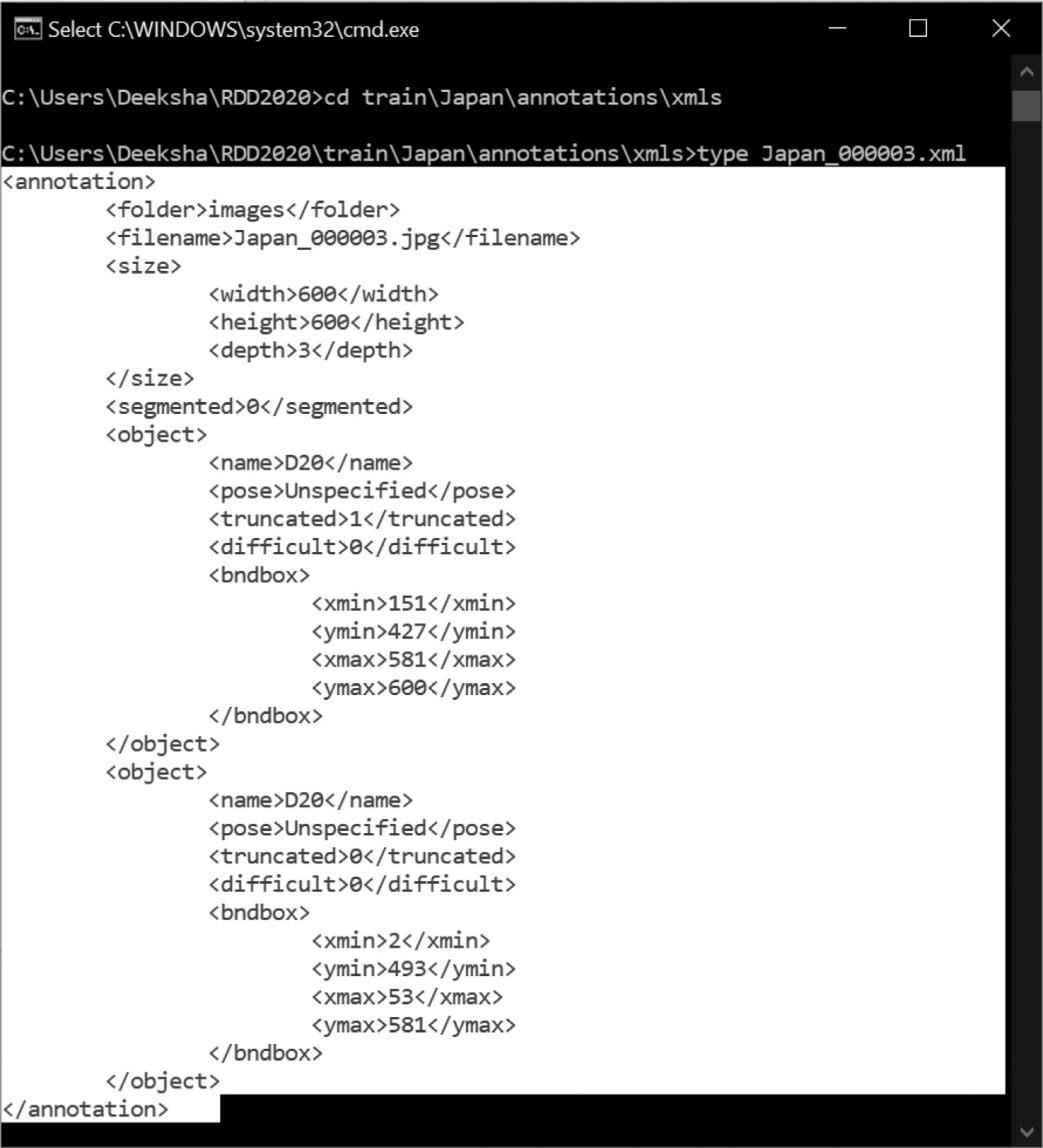
Sample XML file for an image with alligator cracks (D20).

Fig. 6 shows a sample XML file for an image containing alligator cracks (D20) at two locations as specified by the corresponding rectangular coordinates. The annotation pipeline to create the XML files is covered in the following section. The label map containing the class labels for the damage categories is provided as .pbtxt file.

The test1 and test2 directories respectively contain 1313 and 1314 images from Japan, 969 and 990 images from India, and 349 and 360 images from the Czech Republic. The resolution of images in test1 and test2 is the same as those in the train directory. The test1 and test2 images are intended for the evaluation of models trained using provided train data. Hence, the corresponding XML files have not been released. The users need to predict the damage instances in test images using their proposed model. The predicted output may then be evaluated using the leader-boards available on the GRDDC website [6]. The leader-boards are embedded with the evaluation scripts based on ground truth annotation files for the test images. The criteria for evaluation are provided in the research article [5].

## 2 Experimental Design, Materials and Methods

The data collection involves capturing road images using a Smartphone mounted on a moving vehicle. A smartphone application was designed, and road images were captured at the rate of one image per second to photograph images while traveling on the road without leakage or duplication when the average speed of the vehicle is approximately 40 km/h (or 10 m/s). The installation setup of the smartphone in the car is same as used by the authors in [4].

Firstly, 9053 road images were captured from Japan in 2018[7]. The aforementioned Japanese dataset was augmented in 2019 using the Generative Adversarial Network [8] and in 2020 using images from India and Czech Republic [4]. The collected images were annotated in PASCAL VOC format [3] using the labelImg tool. The annotations include marking the road damage label and location in the image. The damage categories for annotating Japanese data are defined using the Japanese Road Maintenance and Repair Guidebook 2013. Accordingly, eight damage categories have been considered for annotating images collected from Japan [7].

**Fig. 7:**
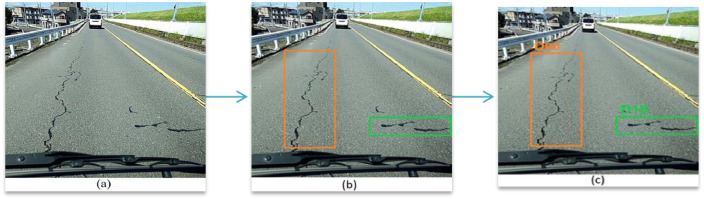
Annotation Pipeline (a) original image, (b) image with bounding boxes, (c) final annotated image containing bounding boxes and class labels.

However, the road standards for evaluations of Road Marking deterioration such as Crosswalk or White Line Blur differ significantly across different countries. Thus, these categories were excluded from the annotations for images collected from India and Czech so that generalized models can be trained applicable for monitoring road conditions in more than one country. The annotation pipeline is shown in Fig. 7. A summary of several state-of-the-art deep-learning models trained using the RDD2020 dataset for global road damage detection is presented in [5].

## CRediT Author Statement

**Deeksha Arya:** Data curation, Methodology, Software, Writing – original draft; **Hiroya Maeda:** Data curation, Methodology, Software, Writing – Review & Editing; **Sanjay Kumar Ghosh:** Supervision, Writing – Review & Editing; **Durga Toshniwal:** Supervision, Writing – Review & Editing; **Yoshihide Sekimoto:** Supervision, Writing – Review & Editing.

## Declaration of Competing Interest

The authors declare that they have no known competing financial interests or personal relationships which have, or could be perceived to have, influenced the work reported in this article.
